# Montreal Veterinary Medical Association

**Published:** 1896-03

**Authors:** Harry H. Dell

**Affiliations:** Secretary-Treasurer


### MONTREAL VETERINARY MEDICAL ASSOCIATION.

A REGULAR meeting of the Association was held in the Library on December 19, 1895, the Honorary President, Dr. D. McEachran, in the chair. Owing to the proximity of the holiday season the attendance was somewhat less than usual. The minutes of the previous meeting having been read and confirmed, Mr. C. H. Higgins requested that the Secretary be instructed to secure for the Library the Experiment Station *Record*, also the reports of the Bureau of Animal Industry of the United States Department of Agriculture.

Mr. J. Anderson Ness, on behalf of the '96 Journal Club, reported some interesting and successful experiments with barium chloride as a therapeutic agent in the treatment of colic. Observations on several cases were reported in detail, which added interest to the report.

Mr. Fred. W. Kee then presented a paper on "Actinomycosis Bovis." Inasmuch as the disease is either increasing or is more promptly recognized than formerly, the choice of the subject was a fortunate one, and the essayist dealt with the matter in a most pleasing manner. Particular emphasis was directed to the fact that many diseased animals which would be rejected as unfit for food in the larger cities, where a system of veterinary inspection exists, are nevertheless placed upon the markets elsewhere, and ultimately find their way into the food-supply. A lengthy discussion followed the reading of the paper, the essayist ably defending his position. Dr. Adami favored the meeting with some remarks on the nature of the disease in man, while Dr. McEachran referred to the disease as manifested among range-cattle in the Northwest.

Mr. E. H. Morris reported a rare case of surgical interference. Called to see a colt which, as the result of an accident, had lacerated one of the carotids, he applied a ligature to the vessel, and eventually was rewarded by a complete recovery, the nutritition of the area supplied by the vessel being effected by the collateral circulation.

Members were appointed to serve on the Experimental Committee, the essayists for the next meeting notified, and adjournment took place.

THE Association met in the Library of the Faculty of Comparative Medicine, January 15th. The chair was taken and meeting called to order by the President, Dr. Baker. Roll-call showed a good attendance of members. The minutes of the previous meeting were read and adopted.

It was decided to add to the Library the *Journal of the American Public Health Association* and also those of the Paris Health Department.

Mr. Harry Dell furnished the case-report for the evening. The case was one of interest on account of its comparative rarity. The subject, an aged deerhound bitch, was brought to the hospital in a dying condition, with no history of previous illness, the only symptom having been noticed being anorexia. Was declared by the man in charge to be well advanced in pregnancy, but physical examination revealed a large amount of fluid as the cause of distention. Death occurred during the night, and the post-mortem brought to light the existence of extensive pathological processes. The most worthy of notice were the presence of a large adeno-carcinomatous growth of the liver, metastases in the lungs, peritonitis, and ulceration of the duodenum. In the discussion following Mr. Higgins stated that curiously enough, Dr. Martin, in an autopsy on a deerhound a few days previously, had encountered a similar condition of the duodenum. This animal had been chloroformed on account of the existence of chorea.

Mr. S. Macnider presented a paper on "Navicular Arthritis and Neurectomy," describing the nature, symptoms, and course of the disease and the indications for operation. A question as to the value of frog-setons in the surgical treatment in this disease arose out of the discussion following, it being in the opinion of many of the members that if an affected animal be given the rest and hygenic surroundings that are necessary after the insertion of setons, but omitting the latter, the results would be as favorable.

Regarding neurectomy it was said to be a palliative treatment, and the feet of the animals, after the operation, should receive particular attention for obvious reasons.

Mr. J. J. McCarry promised a paper on "Post-mortems," and Mr. J. Anderson Ness will furnish the case-report for the next meeting.

The members of the Experimental Committee were then appointed and the meeting adjourned.

HARRY H. DELL,
Secretary-Treasurer.

---

